# Syndromic Testing in Clinical Microbiology and Infectious Diseases: An Updated Review of Diagnostic Performance, Clinical and Healthcare Impact, and Emerging Developments

**DOI:** 10.3390/microorganisms14102214

**Published:** 2026-10-02

**Authors:** Guiqing Wang, Fei Chen

**Affiliations:** Department of Pathology, New York University Grossman School of Medicine and NYU Langone Health Medical Center, New York, NY 10016, USA

**Keywords:** syndromic testing, multiplex PCR, NAAT, antimicrobial stewardship, diagnostic stewardship, respiratory panel, pneumonia, bloodstream infection, gastrointestinal panel, meningitis/encephalitis panel, joint infection, tropical fever, antimicrobial resistance

## Abstract

Syndromic testing, defined as the simultaneous nucleic acid amplification-based detection of multiple potential pathogens and antimicrobial resistance (AMR) determinants directly from clinical specimens, has transformed laboratory diagnosis of infectious diseases in the United States over the past two decades. In contrast to traditional microbiology workflows that rely on culture, phenotypic susceptibility testing, and sequential single-target assays with turnaround times of 24–72 h or longer, syndromic multiplex panels provide comprehensive pathogen identification within 1–5 h, enabling timely clinical decision-making, targeted antimicrobial therapy, and improved patient outcomes. In this review, the current landscape of FDA-cleared syndromic testing panels across seven major clinical syndromes is examined, including respiratory tract infections, pneumonia, bloodstream infections, gastrointestinal infections, meningitis/encephalitis, joint and bone infections, and global febrile/travel-related illnesses. For each syndrome, commercially available panels in the United States are summarized, with emphasis on diagnostic performance, clinical applications, and limitations. Available evidence demonstrates that syndromic testing, when integrated with institutional diagnostic and antimicrobial stewardship programs, can reduce time to optimal therapy and unnecessary antimicrobial use and, in selected settings, shorten hospital length of stay. A mortality benefit has been reported in some patient populations, particularly those with bloodstream infections. Nevertheless, rapid turnaround time and earlier pathogen identification do not necessarily translate into timely clinical action and improved patient outcomes. Challenges related to implementation and optimization of appropriate panel utilization, including evolving AMR epidemiology, dynamic and geographically variable pathogen distributions, the critical role of diagnostic and antimicrobial stewardship, and emerging developments for diagnosis and management of infectious disease, are also discussed.

## 1. Introduction

### 1.1. Evolution of Infectious Disease Diagnostics

Infectious diseases remain among the leading causes of morbidity and mortality worldwide despite major advances in vaccination, antimicrobial therapy, infection prevention, and public health measures [1]. For more than a century, clinical microbiology laboratories have relied primarily on microscopic examination and culture, followed biochemical identification and phenotypic antimicrobial susceptibility testing (ID/AST), and serology for pathogen detection. These methodologies established the foundation of modern infectious disease diagnosis and remain the cornerstone of clinical microbiology. Conventional culture-based techniques offer important advantages, including recovery of viable organisms for AST, epidemiologic typing, and further characterization. However, these methods frequently require prolonged incubation periods, are susceptible to the effects of prior antimicrobial exposure, and may demonstrate limited sensitivity for fastidious, slow-growing, intracellular, or uncultivable organisms [2,3].

The laboratory diagnosis of infectious diseases has undergone a profound transformation over the past three decades in the US. First, automated bacterial identification and susceptibility systems, such as FDA-cleared VITEK 2 (bioMérieux), MicroScan WalkAway (Beckman Coulter), and Phoenix (BD), reduced hands-on time and standardized AST results. Then, the introduction of matrix-assisted laser desorption ionization time-of-flight mass spectrometry (MALDI-TOF MS) in the early 2010s in many US clinical laboratories represented a major advance in speed and accuracy of microbial identification [4,5]. MALDI-TOF enables species-level identification of microbes from isolated colonies within minutes [4]. In addition, the development of point-of-care testing (POCT) devices, initially for rapid influenza and streptococcal antigen detection, demonstrated that clinically relevant results could be delivered within 15–30 min at or near the patient’s bedside. Nevertheless, both phenotypic ID/AST and MALDI-TOF still depend on prior culture growth with prolonged turnaround time (TAT), while antigen-based POCT assays suffer from limited sensitivity.

The molecular revolution that began in the late twentieth century fundamentally transformed clinical microbiology and infectious disease diagnosis. The introduction of polymerase chain reaction (PCR) enabled rapid, highly sensitive, and highly specific detection of microbial nucleic acids directly from clinical specimens. Subsequent advances in nucleic acid extraction technologies, real-time PCR, multiplex amplification, broad-range PCR and sequencing, and laboratory automation progressively expanded the clinical utility of molecular diagnostics. More recently, these developments were further complemented by innovations in next-generation sequencing (NGS), metagenomic sequencing, digital PCR, and artificial intelligence [6,7,8,9]. In parallel, a major transition from antigen-based testing and phenotypic ID/AST to nucleic acid amplification test (NAAT)-based tests has occurred, resulting in substantially improved diagnostic performance and an expanded range of detectable pathogens [3,5].

Among these advances, syndromic testing has emerged as one of the most clinically impactful innovations in infectious disease diagnostics [10,11,12,13]. Unlike conventional targeted molecular assays, which require clinicians to predict the most likely pathogen and order individual tests accordingly, syndromic panels simultaneously evaluate multiple common pathogens associated with a specific clinical presentation using a single specimen and workflow.

### 1.2. Definition and Concept of Syndromic Testing

Syndromic testing refers to multiplex molecular diagnostic assays for simultaneous detection of multiple pathogens associated with a given clinical syndrome from a single clinical specimen, using a single workflow, within a clinically actionable timeframe [10,11,12]. The syndromic testing approach differs fundamentally from culture-based conventional microbiology stepwise, sequential reflex testing workflow for microbial identification and antimicrobial susceptibility testing (Figure 1). Unlike targeted single-pathogen PCR assays, syndromic panels are designed around clinical presentation rather than a suspected specific pathogen. This paradigm shift reflects the recognition that many infectious syndromes (e.g., community-acquired respiratory illness, sepsis, diarrhea, meningitis) are caused by diverse pathogens that produce overlapping clinical manifestations, but the causative pathogen cannot be reliably distinguished on clinical presentations alone. And the delayed microbial ID/AST translates directly into empirical broad-spectrum antibiotic exposure, attendant risks of toxicity, selection pressure for resistance, and when initial empirical choices are inadequate, excess morbidity and mortality in many cases of patients with sepsis, pneumonia, or central nervous system infections [14,15,16].

Most commercial syndromic panels employ multiplex PCR, microarray-based hybridization, or other NAAT platforms to detect a broad array of bacterial, viral, fungal, and/or parasitic pathogens plus selected AMR determinants from a single clinical specimen processed in a cartridge or pouch. This broad, syndrome-driven approach offers several advantages over conventional microbiology and single-targeted PCR assays (Table 1).

**Table 1:** Comparison of conventional microbiology, targeted single-pathogen PCR, and syndromic panel testing *.

Parameter	Conventional Microbiology (Culture + Phenotypic ID/AST)	Targeted Single-Pathogen PCR	Multiplex Syndromic Testing Panel (NAAT)
Testing approach	Stepwise, sequential testing	Single test aligned to suspected pathogen	Single test aligned to clinical syndrome
Specimen type & volume	Any, multiple (1–10 mL)	Various, single (0.2–1 mL)	Various, single (0.2–1 mL)
Test performance			
Pathogens/AMR detectable	1 to 3	1 to 3 targets	6 to 40+ targets simultaneously
Culture-negative/fastidious pathogen detection	No	Yes (single target only)	Yes (broad range)
Specimens collected after antibiotic initiation	Low	High (single)	High (multiple)
Detection of viral pathogen	Low	High (single virus)	High (multiple viruses simultaneously)
Detection of multiple targets (polymicrobial)	Low (viral culture required)	No	Yes (simultaneous multi-target)
Phenotypic MIC/full AST	Yes (24–48 h, gold standard)	No	No (AMR genes only; phenotypic confirmation required)
Genotypic susceptibility	No	Limited (1–2 genes)	5–20 resistance genes per panel
Organisms outside listed panel target	Yes (any cultivable organism)	No	No (panel-defined list only)
Turnaround time (TAT)	24–72+ h (culture → ID → AST)	1–5 h (sample → ID)	1–5 h (sample → ID/Selected AMR)
Complexity & operator skill required	High	Moderate	Moderate (automated, sample-to-answer)
Cost (reagent and capital)	Low–moderate	Moderate	High
Clinical and public health impact			
Antibiotic stewardship impact	Possible after 24–72+ h	Limited	High, broad guidance within 1–5 h
Clinical impact	Delayed diagnosis and targeted therapy	Moderate	Earlier diagnosis and targeted therapy
Infection control/public health impact	Yes (delayed)	Yes (limited)	Yes (rapid, same day cohorting)
Major advantages	Recovery of viable organisms for AST; Enables comprehensive phenotypic characterization and epidemiologic investigations; Supports downstream testing (e.g., serotyping, sequencing, strain typing).	Rapid TAT; High analytical sensitivity and specificity for targeted pathogens; Straightforward result interpretation.	Rapid TAT; High analytical sensitivity and specificity of multiple targets; Simultaneous pathogen identification and AMR detection facilitate timely antimicrobial optimization (escalation, de-escalation, or avoidance of unnecessary therapy); Supports diagnostic and antimicrobial stewardship, and infection prevention practice; Improves clinical management and healthcare system efficiency.
Limitations	Prolonged TAT, potentially delaying targeted therapy; Pathogens may not be recovered; Reduced sensitivity for fastidious organisms and specimens collected after antibiotic initiation.	Detects only requested target(s); May miss co-infections; Cannot distinguish nucleic acids from viable and nonviable organisms and differentiate colonization from infection.	Off-panel pathogens are not detected; Not all AMR mechanisms are identified; Cannot distinguish nucleic acids from viable and nonviable organisms, and colonization vs. infection; Potential for over-detection, inappropriate utilization and interpretation without diagnostic stewardship; High reagent and capital costs.

* ID/AST: Identification/Antimicrobial susceptibility testing; MIC: Minimum inhibitory concentration; AMR: Antimicrobial resistance; NAAT: Nucleic acid amplification test.

Several published reviews summarized the organisms and AMR targets detected by different syndromic panels, their performance and clinical utilization based on earlier available data [10,11,12,13]. This article was designed as a narrative review rather than a systematic review or meta-analysis. A PubMed search was conducted through June 2026 using the search strategy “(syndromic testing panel OR multiplex PCR panel OR BioFire) AND infectious disease,” yielding approximately 1000 records. Approximately 570 publications were reviewed for relevance, more than 60% of which were published within the preceding five years. Publications addressing the diagnostic performance, clinical applications, clinical and healthcare impact, limitations, and implementation of FDA-approved or -cleared syndromic testing panels in the US were prioritized, including relevant studies conducted both within and outside the United States. Relevant professional guidelines, regulatory information, and manufacturer package inserts were additionally reviewed where appropriate. The objective of this review is to provide a state-of-the-art overview of U.S. FDA-approved or cleared syndromic testing panels with 6 targets or more in contemporary infectious disease diagnostics, clinical management, infection prevention and control, and public health across major infectious disease syndromes. Implementation challenges, diagnostic and antimicrobial stewardship considerations, economic implications, and emerging technological developments that are expected to shape the next generation of infectious disease diagnostics are also discussed.

## 2. Syndromic Testing Panels and Applications

Syndromic testing in the U.S. began with FDA clearance of the Luminex xTAG Respiratory Viral Panel (RVP) in 2008, the first broadly multiplex molecular assay for simultaneous detection of 12 respiratory viral pathogens [17]. Subsequent introduction of the FilmArray Respiratory Panel in 2011 transformed syndrome testing into routine clinical practice and accelerated widespread clinical adoption through its integrated sample-to-answer workflow and rapid turnaround time [10]. Since then, syndromic testing has expanded into multiple areas of infectious disease diagnostics. One of the most important factors to drive the widespread and expansion of syndromic testing is the increasing complexity of infectious disease management. Modern healthcare systems care for growing numbers of immunocompromised patients, transplant recipients, oncology patients, and critically ill individuals in whom rapid diagnosis is particularly important. Delays in identifying infectious etiologies may result in prolonged empiric antimicrobial exposure, increased healthcare costs, adverse drug events, and poorer patient outcomes [2,10,18]. In addition, the emergence of antimicrobial resistance, and increasing recognition of healthcare-associated infections, emerging infectious diseases, and global outbreaks has further increased the importance of timely and accurate microbiological diagnosis [5].

Currently available commercial syndromic platforms encompass respiratory pathogen panels (RP), pneumonia panels (PN), bloodstream infection and sepsis panels (BSI), gastrointestinal (GI) pathogen panels, meningitis and encephalitis (ME) panels, joint infection (JI) panels, and travel/global fever (TF) panels. Table 2 summarizes currently FDA-cleared commercial syndromic testing panels and their major characteristics.

### 2.1. Respiratory Pathogens Panel

Acute respiratory tract infections (ARTIs) are among the most common infectious diseases worldwide and remain a leading cause of outpatient visits, emergency department encounters, hospitalizations, and infectious disease-related mortality [19]. The etiologic spectrum is broad, encompassing viruses (influenza A and B, RSV, rhinovirus/enterovirus, parainfluenza virus [PIV] 1–4, adenovirus, hMPV, endemic coronaviruses OC43, HKU1, NL63, and 229E, and SARS-CoV-2) as well as bacteria (*Mycoplasma pneumoniae*, *Chlamydia pneumoniae*, *Bordetella pertussis*, and *Bordetella parapertussis*), and clinical features overlap substantially across etiologies [19,20]. One of the greatest challenges in respiratory infectious disease management is the substantial overlap in clinical manifestations among viral and bacterial pathogens [19,20]. Traditional respiratory diagnostic testing relied on viral culture, direct fluorescent antibody testing, rapid antigen detection assays, and targeted molecular tests. While these methods remain useful in selected circumstances, they possess important limitations related to sensitivity, specificity, TAT, and pathogen coverage. This diagnostic uncertainty often drives extensive and unnecessarily prolonged empirical antibiotic treatment, as clinicians cannot reliably distinguish viral from bacterial infections among these patients.

Respiratory pathogen panels represent one of the most mature and widely implemented categories of syndromic testing. They serve as a model for the broader application of multiplex molecular diagnostics across numerous infectious disease syndromes. Multiple FDA-approved or cleared respiratory syndromic panels are commercially available in the U.S., with target coverage ranging from 6 to more than 20 viral and bacterial pathogens and typical TAT of 1 to 5 h (Table 2). Among the most widely adopted assays is the FilmArray Respiratory Panel (RP 2 and RP2.1; bioMérieux/BioFire), which detects a broad spectrum of respiratory viruses and atypical bacterial pathogens directly from nasopharyngeal swabs with a TAT of approximately one hour [21]. It is estimated that the FilmArray platforms are currently utilized by nearly 70% of US clinical laboratories. Other commonly used comprehensive respiratory syndromic panels in the U.S. include the ePlex Respiratory Pathogen Panel (RPP; Roche/GenMark) [22], NxTAG Respiratory Pathogen Panel (DiaSorin/Luminex) [23] and QIAstat-Dx Respiratory Panel (Qiagen) [24], all of which provide broad multiplex detection of respiratory pathogens. Comparative studies have consistently demonstrated excellent analytical performance across currently available multiplex respiratory panels. Overall positive and negative percent agreement values typically range from 94.1% to 99.5%, indicating comparable diagnostic performance among the major commercial assays [10,23,24,25,26]. None of the current respiratory panels include AMR targets, as respiratory tract bacterial infections detectable by these panels (atypical bacteria, *Bordetella* spp.) do not routinely harbor the clinically relevant AMR determinants captured on pneumonia or BSI panels.

In addition, several focused multiplex respiratory panels with five or fewer but clinically targeted analytes are also available in U.S. clinical laboratories and point-of-care settings [27,28]. These panels are not discussed further in this review, which focuses on multiplex syndromic panels comprising six or more targets.

Respiratory syndromic testing panels may substantially increase pathogen detection compared with conventional diagnostic [10,29]. They are particularly valuable among hospitalized patients, emergency department populations, immunocompromised hosts, and pediatric patients. In these settings, the clinical utility of respiratory syndromic testing extends beyond improved analytical performance. Furthermore, rapid pathogen identification influences several aspects of patient management, ASP and infection control practices, including lower odds ratio for admission, timely antiviral therapy and shorter duration of antibiotic administration, patient cohorting and isolation decisions, and need for additional diagnostic testing [29,30,31,32].

Syndromic testing of patients with upper RTIs allows rapid detection and differentiation of multiple viral versus bacterial respiratory pathogens, supports timely initiation of antivirals, targeted therapy or focused support care, while reducing unnecessary antibiotic exposure and adverse events [12].

Systematic review and meta-analyses demonstrate the high accuracy of multiplex syndromic panels, a reduction in time to results and length of stay for patients overall along with improvements in appropriate antiviral and infection control management among influenza-positive patients, which supports the use of rapid sample-to-answer multiplex PCR testing for respiratory viruses in the hospital setting [26,33]. In a meta-analysis with 27 studies and 17,321 patient encounters, rapid multiplex PCR testing was associated with a reduction of ~24 h (95% CI −28.7 to −19.4 h) in the time to results. Hospital length of stay was decreased by 0.82 days (95% CI −1.52 to −0.11 days). In addition, antivirals were more likely to be given (RR 1.25, 95% CI 1.06–1.48) and appropriate infection control facility use was more common with rapid multiplex PCR testing (RR 1.55, 95% CI 1.16–2.07) among influenza positive patients [33].

Limitations of current respiratory panels include incomplete pathogen coverage, inability to provide comprehensive antimicrobial susceptibility information, relatively high testing costs, the inability to distinguish upper respiratory colonizers from true pathogens (particularly in immunocompromised hosts). Respiratory panels detect nucleic acids rather than viable organisms and therefore may identify pathogens in asymptomatic individuals during prolonged shedding following recovery, or in situations where the detected organism is not responsible for the patient’s current symptoms. This issue is particularly relevant for rhinoviruses, adenoviruses, and certain seasonal coronaviruses, which may be detected for extended periods following clinical recovery. Therefore, syndromic testing results should always be interpreted in clinical context.

### 2.2. Pneumonia Panel

Lower respiratory tract infections including pneumonia remain among the top 10 leading causes of death across all age groups worldwide and are the most common cause of infection-related mortality [1]. A wide array and increasing number of microbial agents can cause various pneumonia syndromes [34,35]. Of these, community-acquired pneumonia (CAP) represents the fifth most common and second most costly reason for hospitalization among children [36], while hospital-acquired pneumonia (HAP) and ventilator-associated pneumonia (VAP) are among the most serious and costly healthcare-associated infections, with attributable mortality rates of 20–30% among mechanically ventilated ICU patients [34,36,37].

Current laboratory diagnosis of pneumonia relies on multiple methods. Molecular methods are most used for the detection of viral pathogens and many atypical bacteria, whereas culture remains the gold standard for the diagnosis of bacterial pneumonia. The sensitivity of culture for detecting different bacterial pathogens, as well as result interpretation and reporting, varies among and within institutions. In addition, bacterial culture with organism ID/AST typically requires 48–72 h before actionable results become available, resulting in prolonged empirical broad-spectrum antimicrobial therapy with agents such as carbapenems, piperacillin-tazobactam, and anti-MRSA agents, thereby potentially driving antimicrobial resistance.

Currently available syndromic pneumonia panels are listed in Table 2. The FilmArray Pneumonia (PN) Panel and Pneumonia Plus (bioMérieux/BioFire), cleared by the FDA in 2018, are the most comprehensive and widely adopted panels in the U.S. The FilmArray PN Panel detects 33 targets (18 bacteria, 8 respiratory viruses, and 7 AMR genes) with a TAT of approximately 1 h from BAL, sputum, and tracheal aspirate specimens, while the Pneumonia Plus panel additionally includes Middle East respiratory syndrome coronavirus (MERS-CoV) [38,39,40]. The Unyvero LRT (OpGen/Curetis) is another FDA-cleared syndromic panel for pneumonia that detects 30 targets (19 bacteria, *Pneumocystis jirovecii*, and 10 AMR genes) but does not include any viral pathogens [41]. It initially received FDA De Novo clearance for testing endotracheal aspirates in 2018 and later received 510(k) clearance for BAL fluid specimens in 2019. Currently, very few U.S. clinical laboratories use the Unyvero LRT panel, possibly in part because of its lack of viral targets and longer TAT of approximately 5 h, compared with approximately 1 h by the FilmArray PN Panel.

The performance of pneumonia syndromic panels has been evaluated in several clinical studies involving both adult and pediatric patients [38,40,41,42]. A multicenter clinical trial analyzing 846 prospectively collected BAL specimens and 836 sputum specimens using the FilmArray PN panel demonstrated an overall positivity rate of 48.8% for BAL and 72.0% for sputum specimens [38]. The sensitivity of this assay was >95% for 10 targets in both BAL and sputum specimens. Sensitivities for the other five organisms ranged from 75% to 91.7%; specificity for all targets in both specimen types was >91% [38]. Compared with routine microbiological methods, the FilmArray PN panel detected significantly more pathogens in both BAL (57.1% vs. 23.1%) [40] and sputum (91% vs. 60%) specimens [43]. It also identified multiple targets in >25% of BAL and sputum specimens, including two or more bacterial organisms or mixed bacterial and viral pathogens [38]. In addition, the FilmArray PN panel reports semi-quantitative bacterial targets from 10^4^ copies/mL to ≥10^7^ copies/mL; organisms reported at ≥10^5^ copies/mL correlated well with conventional culture-positive, which may help in discriminating true lower respiratory pathogens from potential colonizers. One meta-analysis showed that values ≥10^7^ copies/mL are consistently associated with a high probability of culture positivity, regardless of the respiratory sample type and the bacterial target considered, whereas lower values are less frequently associated with positive culture [44]. It is noteworthy that the semi-quantitative values by the FilmArray PN panel are consistently higher than the bacterial loads from quantitative cultures, with pooled mean differences of 1.17 and 0.95 log10 copies/mL for BAL and sputum specimens, respectively, per a recent meta-analysis [45]. Also, these semi-quantitative results should be interpreted together with specimen quality, clinical findings, Gram-stain and culture results, and other laboratory data and should not be used independently to distinguish colonization from infection. It has been a common practice to screen the quality of sputum specimens by Gram-stain before performing the respiratory syndromic panels in many U.S. clinical laboratories, although a recent study in Norway suggests that all sputum samples submitted for rapid syndromic PCR testing should be analyzed, regardless of initial microscopy quality assessment [46].

The currently available PN panels provide rapid TAT, increased pathogen detection, and simultaneous detection of bacterial and viral pathogens, multiple-target detection, and common AMR markers. These analytical advantages are expected to translate into several clinical benefits. One of the most important clinical benefits is the antibiotic optimization, particularly in critically ill patients with pneumonia, through more timely antibiotic escalation and de-escalation [40,42,47,48].

A randomized controlled trial conducted at the Mayo Clinic (Rochester, MN) demonstrated that clinical implementation of the FilmArray PN panel resulted in more rapid antibiotic escalation, including therapy directed against Gram-negative and Gram-positive bacteria, as well as faster de-escalation of Gram-positive antimicrobial therapy [47]. The median time to appropriate antibiotic escalation was reduced by approximately 12 h in patients with CAP [47,49]. No significant benefits were observed with respect to hospital length of stay, 30-day or 90-day mortality, or readmission rates [49]. In another randomized trial of hospitalized patients with community-acquired pneumonia, Abelenda-Alonso et al. found that multiplex PCR testing did not significantly reduce the primary outcome of days of antibiotic therapy compared with conventional microbiological testing (difference, −1.04 days; 95% CI, −2.42 to 0.17) [39]. More recently, the multicenter INHALE WP3 randomized trial in patients with suspected hospital- or ventilator-associated pneumonia demonstrated improved appropriate and proportionate antimicrobial prescribing at 24 h with rapid ICU-based syndromic PCR (76.5% vs. 55.9%); however, non-inferiority for clinical cure at 14 days was not demonstrated (56.7% vs. 64.5%) [50]. Available evidence from clinical trials and meta-analyses indicates that, although rapid syndromic testing can improve antimicrobial stewardship, these improvements may not consistently translate into better clinical outcomes, and evidence for reduced in-hospital mortality among critically ill patients with pneumonia remains limited [49,50,51].

Limitations of current PN panels include the absence of some off-panel pathogens and the lack of complete antimicrobial susceptibility data. A recent study of ICU patients with suspected HAP or VAP reported a 12% false-negative rate for the FilmArray PN panel; therefore, a negative PN panel result does not exclude pneumonia, especially in patients infected with non-targeted Gram-negative bacteria or those with recent antibiotic exposure [52]. Although the semi-quantitative bacterial load results generated by the FilmArray PN panel may aid in interpreting the clinical significance of detected targets, differentiating true pathogens from colonizing organisms, or nucleic acids originating from viable versus nonviable organisms in the respiratory tract, remains challenging and may potentially lead to overtreatment. Another limitation of the Unyvero LRT panel is its restriction to bacterial targets; it must be combined with a separate viral NAAT if respiratory viral co-infection is clinically suspected.

### 2.3. Bloodstream Infection Panel

Bloodstream infection (BSI) and sepsis are major global causes of morbidity and mortality. Each year, an estimated 48 million people develop sepsis, resulting in ~11 million deaths worldwide [18,53,54]. The Surviving Sepsis Campaign guidelines recommend obtaining blood cultures and initiating empiric antibiotics within one hour of sepsis recognition as each hour of delay in appropriate antibiotic therapy has been associated with a measurable increase in mortality [55]. Conventional blood culture systems typically require 14–72 h for positivity signal, and full organism identification with susceptibility testing adds another 24–48 h, for a total TAT of 24–72 h before optimized targeted therapy can be initiated [12,56].

Syndromic panels applied to positive blood culture bottles include the FilmArray BCID2 Panel (33 organism targets and 10 AMR genes, bioMérieux BioFire), the ePlex BCID-GP/GN/FN Panel trio (modular 23/27/15 target panels covering Gram-positive, Gram-negative, and fungal organisms and AMR genes, respectively, Roche/GenMark), the Verigene Positive Blood Culture Test (BC-GP, BC-GN)) and LIAISON Plex Yeast Blood Culture Assay (DiaSorin/Luminex). Direct whole-blood panels without blood culture prerequisite include the T2Bacteria Panel (6 targets) and T2Candida Panel (5 targets each; TAT 3–5 h, T2 Biosystems).

Syndromic panel testing on positive blood culture can provide rapid and accurate identification of more than 40 common BSI organisms and AMR markers with an overall sensitivity of ~98% and specificity of ~99% from approximately 90% of positive blood culture bottles, leading to accelerated optimal therapy, enhanced antimicrobial stewardship and improved patient outcomes [16,57,58].

The clinical impact of BSI panels has been demonstrated in multiple clinical studies and meta-analyses [58,59,60,61]. An early randomized clinical trial demonstrated that rapid multiplex PCR performed on positive blood cultures, when combined with active ASP intervention, significantly reduced the median time to effective antimicrobial therapy and decreased the use of broad-spectrum antibiotics compared with standard care [59]. Two meta-analyses further demonstrated that implementation of rapid molecular identification was associated with shorter time to optimal antimicrobial therapy, reduced hospital length of stay, and lower mortality among patients with bloodstream infections [16,58]. Of these, the most comprehensive meta-analysis by Peri et al. (2024), which included 88 studies and 25,682 patient encounters, showed that rapid molecular identification combined with concurrent ASP intervention achieved the greatest reduction in time to appropriate antimicrobial therapy (18–29 h) and was associated with significant reductions in both hospital length of stay and all-cause mortality [16]. A more recent systematic review and meta-analysis, including 20 studies and 4587 patients with BSI across U.S. hospitals, showed that BCID implementation was associated with a 17.28-h reduction in time to appropriate antimicrobial therapy and a 1.25-day reduction in hospital LOS; however, no statistically significant reduction in mortality was observed [62].

Evidence supports the routine use of BSI panels as an emerging standard of care for BSI and sepsis management [15,16,58]. Nevertheless, syndromic panels are adjunct to, not replacement for, standard blood culture methods to identify pathogens not included in the panel and to provide comprehensive, phenotypic AST results. Current panels are dependent on positive blood culture and have only incomplete pathogen and AMR gene coverage. False positives and false negatives in detecting BSI organisms and predicting resistance in isolates with other independent resistance mechanisms are also documented [12,63]. In addition, its clinical benefits are largely relied on an integrated institutional stewardship program [16,58].

### 2.4. Gastrointestinal Panel

Approximately 2–4 billion episodes of acute gastroenteritis occur annually worldwide, making it one of the most common diagnoses in emergency departments globally. Viral, bacterial, and protozoal pathogens often produce clinically indistinguishable syndromes [64]. It is estimated that approximately 179 million cases of diarrhea occur in the US each year [65]. Traditional stool workup using bacterial culture, ova-and-parasite examination, and enzyme immunoassay or PCR for individual targets is time-consuming, has a low diagnostic yield, and often fails to identify the causative pathogen.

The BioFire FilmArray Gastrointestinal (GI) Panel was the first FDA-cleared syndromic gastrointestinal multiplex panel in the US, receiving FDA 510(k) clearance in May 2014, followed by the Luminex xTAG Gastrointestinal Pathogen Panel (GPP) in September 2014. Subsequently, several additional syndromic GI panels, representing a broader range of manufacturers than other syndrome categories, have been cleared by the FDA. These include the QIAstat-Dx GI Panel 2 (Qiagen, 2024), Panther Fusion GI Panels (Hologic, 2025), Xpert GI Panel (Cepheid, 2026), and the LIAISON PLEX Gastrointestinal Flex Assay (DiaSorin, 2026) (Table 2). The QIAstat-Dx GI Panel 2 detects 16 targets with a comparable ~1-h TAT to the FilmArray GI Panel [66,67]. The Xpert GI Panel detects 11 targets selected to maximize clinical actionability, epidemiological relevance, and impact on patient management while maintaining cost-effectiveness for primary care and outpatient settings. The LIAISON GI Flex assay offers a unique flexible testing capability that allows laboratories to customize which 24 pathogen targets are reported, supporting diagnostic stewardship and potentially reducing unnecessary testing.

The FilmArray GI Panel (bioMérieux/BioFire), which detects 22 gastrointestinal pathogens (13 bacteria, 5 viruses, and 4 parasites), has become one of the most widely adopted multiplex syndromic panels for the diagnosis of infectious gastroenteritis in U.S. [64,68,69,70]. A large multicenter evaluation of the FilmArray GI Panel on 1556 prospectively collected stool specimens in the US demonstrated that had a sensitivity of 100% for 12 of the 22 targets and >94.5% for an additional 7 targets, while the overall specificity exceeded 97.1% for all panel targets [68]. Multiple pathogens were detected in 31.5% of positive specimens [68]. A similar multicenter study in Europe evaluated 709 stool samples using the FilmArray GI Panel and found that 384 (54.2%) were positive for at least one pathogen, compared with 128 (18.1%) positive specimens identified by conventional laboratory methods [71]. Clinical studies have consistently demonstrated that the FilmArray GI Panel substantially increases pathogen detection rates compared with conventional stool culture (overall positivity, 35–54% vs. 6–18%, respectively) (61, 65, 66, 68). A most recent retrospective observational study at 12 U.S. hospitals using the FilmArray GI panel reported the overall positivity rate was 22% among 16,570 adult patients. Enteropathogenic *Escherichia coli* (EPEC) and *Norovirus* were the most common organisms detected (6.4% and 6.1%, respectively). The third most common organism varied based on high-risk comorbidity, typically *Salmonella* or *Campylobacter* [69].

Comparative studies of different FDA-cleared GI panels have demonstrated high diagnostic accuracy and positive percent agreement [66,67,68,72]. Despite its high sensitivity, detection of *C. difficile* requires careful interpretation because the assay detects toxin A/B genes but cannot distinguish active infection from asymptomatic colonization. Therefore, limiting testing to patients with clinically significant diarrhea through diagnostic stewardship interventions is strongly recommended [72,73].

Torres-Miranda et al. (2020) reported that implementation of the FilmArray GI Panel was associated with earlier initiation of optimal antimicrobial therapy, reduced antibiotic use, and a significantly shorter hospital length of stay (median 3.0 vs. 7.5 days) [74]. In another US study, the median time from collection to initiation of antimicrobial therapy was 22 h for FilmArray and 72 h for culture [64]. Beal et al. (2018) demonstrated that use of the FilmArray GI Panel reduced the need for additional diagnostic testing and abdominal imaging and was associated with lower healthcare costs, with an estimated savings of $293.61 per patient, as well as a modest reduction in hospital length of stay [75].

The main challenges in utilizing syndromic GI panels include uncertainty regarding the clinical significance of some detected organisms, particularly enteropathogenic *Escherichia coli* (EPEC). Also, false-positive and false-negative results have been reported and may lead to missed diagnosis or unnecessary infection prevention and control measures [76,77]. In addition, the relatively high assay cost and limited reimbursement have long posed challenges for clinical laboratories implementing these syndromic panels.

Current guidelines do not recommend routine use of syndromic GI panels for all patients with acute gastroenteritis. Instead, multiplex GI panels are recommended for selected patients with severe illness, bloody diarrhea, fever, immunocompromised status, suspected enteric outbreaks, or other clinical and epidemiologic features in which rapid pathogen identification is expected to influence patient management or public health interventions [2,73,78].

### 2.5. Meningitis and Encephalitis Panel

Meningitis and encephalitis (ME) represent some of the most urgent and potentially devastating infectious diseases in clinical medicine. Acute bacterial meningitis, viral meningitis, encephalitis, meningoencephalitis, and fungal CNS infections are associated with substantial morbidity and mortality with an overall inpatient mortality of 2.9% in the U.S, which are higher in those with bacterial (8.2%), fungal (8.2%), or arboviral (8.9%) disease, particularly when diagnosis and treatment are delayed [79]. Consequently, rapid and accurate microbiological diagnosis is critical for optimizing patient outcomes.

Conventional microbiological diagnosis relies on CSF Gram-stain, bacterial culture, fungal culture, antigen testing, serology, and pathogen-specific molecular assays. In practice, clinicians often order multiple individual assays to evaluate a single patient, leading to increased laboratory complexity, prolonged turnaround times, and fragmented diagnostic workflows [80]. Of these, CSF culture has limited sensitivity (50–90% for bacterial meningitis) and TAT of 48–72 h and viral culture is largely abandoned in the US clinical laboratories [80,81].

Two FDA-cleared syndromic ME panels are currently available in the US. The FilmArray ME Panel (bioMérieux/BioFire) was cleared by FDA in 2016. It is the first commercial syndromic ME panel that detects 14 targets (6 bacteria, 7 viruses, 1 fungus) from CSF in ~1 h [81]. The QIAstat-Dx ME Panel (Qiagen) was cleared by FDA in 2024. The FDA-cleared QIAstat-Dx ME panel detects 9 targets (7 bacteria, 1 virus and 1 fungus) compared to its CE-IVD version that detects up to 16 targets [82,83].

The FilmArray ME Panel was evaluated in a multicenter study by Leber et al. (2016) across 13 US institutions (n = 1560 CSF specimens) and demonstrated overall sensitivity of 81–100% and specificity of 96–99.5% across targets, with highest performance for HSV, enterovirus, and *S. pneumoniae* [81]. Restricting FilmArray ME orders in pediatric patients based on pleocytosis or other abnormal parameters may result in missed diagnostic opportunities, particularly for the detection of viruses [84].

The clinical utility of rapid ME syndromic testing extends beyond analytical performance [80,85,86]. Prompt identification of bacterial meningitis pathogens may support early narrowing of empirical antimicrobial therapy, while rapid detection of HSV can facilitate timely initiation or continuation of acyclovir therapy. Similarly, identification of enteroviral meningitis may reduce unnecessary antimicrobial exposure and shorten hospital stays in selected patient populations.

Among immunocompromised patients, rapid detection of pathogens such as *Cryptococcus neoformans* or CMV may significantly influence management decisions. Consequently, CNS syndromic testing has become an important component of diagnostic workflows in many tertiary-care hospitals and academic medical centers.

Current syndromic ME panels remain limited by their predefined pathogen content. As a result, clinically important pathogens not included in the assay, such as *Mycobacterium tuberculosis*, *Aspergillus* spp., *Toxoplasma gondii*, West Nile virus, and JC virus, will not be detected. Consequently, a negative result does not exclude CNS infection, particularly in immunocompromised patients or in regions where these pathogens are endemic. Additional targeted molecular, serologic, microbiologic, or metagenomic testing may therefore be required when clinical suspicion remains high [80]. Positive results also require careful clinical interpretation, particularly for targets with low prevalence, for which the positive predictive value may be reduced. False-positive results have been reported, while false-negative HSV results may occur depending on the timing of CSF collection relative to disease onset. Detection of HHV-6 presents an additional interpretive challenge because viral nucleic acid may reflect latent/reactivated infection or chromosomally integrated HHV-6 rather than active CNS disease. Therefore, discordant results should be evaluated in the context of clinical presentation and, when appropriate, confirmed or supplemented by targeted testing.

### 2.6. Joint Infection Panel

Septic arthritis (SA) and periprosthetic joint infection (PJI) are among the most challenging orthopedic infections. While septic arthritis is a serious and potentially life-threatening infection involving one or more joints, prosthetic joint infection represents one of the most severe and costly complications following total joint replacement (arthroplasty) [87,88,89]. The diagnosis of PJI is established by the presence of a sinus tract communicating with the prosthesis or by two positive cultures yielding the same organism [87,88,90], whereas SA is diagnosed based on clinical presentation, elevated inflammatory biomarkers, and identification of the infecting organism in synovial fluid by culture and/or molecular testing [89,91,92].

The traditional diagnostic workflow includes blood cultures and inflammatory biomarkers, e.g., WBC count with differential, CRP, and ESR, synovial fluid and tissue analysis (including WBC count with differential, Gram-stain, and culture), and diagnostic imaging [12]. Diagnosis typically requires multiple synovial fluid and tissue cultures, with turnaround times ranging from 5 to 14 days and culture positivity rates of only 65–80%, owing to low bacterial burden, fastidious organisms, prior antibiotic exposure, and biofilm formation [12,87,88,92].

The FilmArray Joint Infection (JI) Panel (bioMérieux/BioFire) is the first and currently the only FDA-cleared (2022) commercial syndromic panel for testing synovial fluid in the U.S. The assay simultaneously detects 39 targets (31 organisms and 8 AMR genes) and improves identification of fastidious or difficult-to-culture organisms, including *Kingella kingae*, streptococci, *Neisseria gonorrhoeae*, and anaerobic bacteria [93,94].

Implementation of the FilmArray JI Panel in clinical laboratories has demonstrated a rapid turnaround time (approximately 1 h compared with 2–7 days for culture), with reported sensitivity and specificity of 96.3% and 97.8%, respectively, compared with conventional microbiologic methods [93,95]. A combined diagnostic approach using synovial fluid and tissue culture together with the JI Panel provides the highest diagnostic sensitivity, particularly in culture-negative cases. Current clinical guidelines emphasize synovial fluid culture and conventional microbiological evaluation in the diagnosis of septic arthritis and PJI. Molecular syndromic testing may serve as an adjunct in selected cases, particularly when rapid organism identification could influence management, but it does not replace conventional culture and susceptibility testing [12,90,92].

Rapid and accurate pathogen and AMR detection by syndromic testing of synovial fluid supports earlier surgical and antimicrobial decision-making, facilitates prompt transition from empirical to targeted antimicrobial therapy, and has the potential to improve patient outcomes through reductions in hospital length of stay and complications [93,95]. Additional benefits include reducing the need for further diagnostic testing and costly imaging studies, supporting antimicrobial stewardship through decreased empirical use of broad-spectrum antibiotics, and providing valuable institutional and regional epidemiologic data on joint infection pathogens [12].

Current syndromic testing panels for joint infection remain limited to predefined targets and therefore cannot detect organisms that are absent from the assay design. Consequently, a negative PCR result does not exclude infection or polymicrobial infection involving off-panel organisms. For example, two clinically important causes of prosthetic joint infection, *S. epidermidis* and *Cutibacterium acnes*, are not included in the current FilmArray JI Panel [93]. Likewise, the panel has limited utility for diagnosing chronic arthritis caused by *M. tuberculosis*, nontuberculous mycobacteria (NTM), viruses (e.g., parvovirus B19), and dimorphic fungi. Rare false-positive results may also occur because of target cross-reactivity or specimen contamination. Looking ahead, incorporation of host-response biomarkers and artificial intelligence-assisted interpretation may improve future diagnostic algorithms by distinguishing infection from aseptic inflammation or mechanical prosthesis failure, thereby further enhancing the diagnostic accuracy and clinical utility of molecular testing for orthopedic infections.

### 2.7. Global Fever and Travel-Associated Disease Panels

Acute febrile illness in returned travelers or in endemic tropical regions presents a significant diagnostic challenge. Dengue fever, malaria, chikungunya, leptospirosis, typhoid fever and other travel-associated diseases produce overlapping clinical syndromes, and rapid differentiation is critical for timely appropriate therapy. Traditional diagnostic approaches for acute febrile illness are often fragmented and inefficient. Clinicians frequently order multiple individual tests based on epidemiologic history and clinical suspicion, resulting in prolonged diagnostic evaluations that may take days or weeks to complete.

The FilmArray Tropical Fever (TF) Panel (bioMérieux/BioFire; 6 targets) is the first multiplex panel cleared by FDA in 2024 for acute febrile tropical illness, detecting dengue virus (serotypes 1–4), Chikungunya virus, *Leptospira* spp., and *Plasmodium* species from whole blood with a ~1-h TAT. It has been introduced in US clinical laboratories only until very recently. Another FDA cleared panel, Global Fever Special Pathogen (GFSP) Panel (FDA-cleared 2022; 16 targets, BioFire Defense) expands coverage to include *Bacillus anthracis*, *Chikungunya virus*, *Crimean-Congo hemorrhagic fever virus (CCHF)*, dengue virus, *Ebola virus*, *Francisella tularensis*, *Lassa virus*, *Leishmania* spp., *Leptospira* spp., *Marburg virus*, *Plasmodium* spp. including the differentiation between *P. falciparum* and *P. vivax*/*ovale*, *Salmonella enterica* serovar Paratyphi A, *Salmonella* enterica serovar typhi, *West Nile virus*, *Yellow Fever virus*, *Yersinia pestis*, and the *Zika virus*. Unlike many traditional syndromic assays, the GFSP Panel was specifically designed to address a broad range of pathogens that may present primarily as fever without localizing symptoms. The assay utilizes whole blood specimens and simultaneously evaluates multiple bacterial, viral, and protozoal pathogens associated with travel-related illness, zoonotic infections, and public health emergencies.

A prospective multicenter evaluation of the BioFire TF Panel on 1965 patients from 10 global sites found sensitivity of 97.4% for *Plasmodium* spp., 97.1% for dengue virus, 88.2% for *Chikungunya*, and 81.7% for *Leptospira*, outperforming most conventional comparators [96]. Another prospective clinical evaluation for the GFSP Panel at 11 geographically distinct global sites with 2139 whole blood specimens detected at least one analyte in 33.9% (725/2139) of specimens. The most prevalent analytes were *Plasmodium* (29.0%, 118/407) and dengue virus (36.4%, 266/730). The overall positive and negative percent agreement between GFSP panel and comparator testing ranged between 92.7–100% and 99.2–100%, respectively, suggesting that this syndromic panel can be a valuable tool to aid in rapid diagnosis and national preparedness for disease outbreaks [97].

Rapid diagnosis may facilitate earlier initiation of appropriate therapy for treatable infections such as leptospirosis and selected bacterial zoonoses. Beyond individual patient care, fever syndromic testing possesses substantial public health value. Early recognition of emerging pathogens, imported infections, and outbreak-associated diseases may support epidemiologic investigations and implementation of infection prevention measures.

One of the unique aspects of fever syndromic testing is the importance of laboratory biosafety. Many pathogens included within GFSP are associated with significant occupational risks or require enhanced containment procedures. Consequently, implementation considerations extend beyond routine diagnostic performance and encompass broader laboratory safety requirements.

Interpretation may also be complicated by geographic variability in pathogen prevalence. Diseases commonly encountered in one region may be rare elsewhere, influencing both pretest probability and predictive value. Consequently, epidemiologic history remains a critical component of result interpretation.

## 3. Syndromic Testing Benefits and Impact on Healthcare System

Table 3 summarizes the potential diagnostic and antimicrobial stewardship, infection prevention and control, public health, and economic benefits of currently available syndromic testing panels across a healthcare system.

Syndromic testing has substantially improved the efficiency of clinical microbiology laboratories and enhanced institutional diagnostic stewardship program by enabling simultaneous detection of multiple pathogens and selecting AMR determinants from a single clinical specimen in a single assay. Compared with conventional microbiology workflows that rely on culture, biochemical identification, and phenotypic susceptibility testing, syndromic panels reduce turnaround times from days to hours and provide standardized, highly automated testing processes. These assays also improve the detection of fastidious, nonviable, or difficult-to-culture organisms [10,11,12].

The rapid availability of syndromic testing results has been associated with earlier pathogen-directed clinical management, improved diagnostic confidence, and timely initiation of appropriate antimicrobial therapy, as described for the individual syndromic testing panels. Numerous studies have demonstrated that implementation of syndromic testing is associated with shorter hospital length of stay, reduced mortality, and improved overall patient outcomes in appropriately selected healthcare settings and patient populations, particularly in patients with BSI and sepsis when integrated with clinical decision support and antimicrobial stewardship interventions [14,16,40,98,99].

Syndromic testing has enhanced the feasibility and practice of antimicrobial and diagnostic stewardship [14,16,40,100]. Rapid identification of pathogens and key resistance determinants facilitates earlier optimization of antimicrobial therapy, including escalation, de-escalation, narrowing of the antimicrobial spectrum, or discontinuation of unnecessary treatment. Several studies have demonstrated reductions in broad-spectrum antibiotic utilization, antimicrobial exposure days, and inappropriate prescribing when syndromic testing is combined with active ASP review and intervention. The clinical impact of these assays is generally greatest when laboratory results are integrated into structured antimicrobial stewardship workflows [16].

Syndromic testing has promoted the development and implementation of diagnostic stewardship programs. Appropriate patient selection, specimen collection, and interpretation of multiplex molecular results are essential to maximize clinical benefit while minimizing unnecessary testing and inappropriate antimicrobial use [15,100,101,102,103]. Current IDSA/ASM laboratory utilization guidelines emphasize that syndromic panels should be ordered selectively when the results are expected to influence patient management, infection prevention measures, or public health interventions [2]. Integration of laboratory expertise with institutional diagnostic stewardship programs helps ensure optimal test utilization and appropriate interpretation of multiplex molecular results, particularly for organisms of uncertain clinical significance, asymptomatic colonization, or polymicrobial detections [100].

Early identification of transmissible pathogens enables earlier implementation of infection prevention and control measures, including transmission-based precautions, patient cohorting, and outbreak investigations [33,104,105]. Earlier molecular detection may also reduce nosocomial transmission by shortening the interval between specimen collection and implementation of appropriate containment measures. Respiratory pathogen panels provide a clear example of this benefit. Early identification of influenza virus, respiratory syncytial virus (RSV), or SARS-CoV-2 facilitates timely implementation of transmission-based precautions and supports healthcare resource allocation during seasonal outbreaks. Similarly, gastrointestinal panels enable rapid recognition of norovirus and other highly transmissible enteric pathogens, reducing delays in infection prevention interventions. Bloodstream infection panels contribute to infection prevention through early detection of multidrug-resistant organisms such as MRSA, VRE, and carbapenem-resistant *Enterobacteriaceae* (CRE). Rapid recognition of these pathogens may prompt enhanced infection control measures and facilitate outbreak investigations and epidemiologic surveillance.

Syndromic testing contributes to public health surveillance by enabling earlier identification of seasonal outbreaks, emerging infectious diseases, antimicrobial resistance trends, and enhanced surveillance of circulating pathogens and unusual epidemiologic events [106,107,108]. During the COVID-19 pandemic and other public health emergencies, multiplex molecular platforms demonstrated the value of rapid, scalable diagnostic testing [107,108] in supporting surveillance, outbreak management, and public health decision-making [109]. In addition, cloud-based surveillance networks integrating deidentified results from syndromic testing platforms have been established to provide near real-time institutional, regional, and national surveillance of respiratory, gastrointestinal, bloodstream, and antimicrobial resistance targets [12,108]. These networks facilitate monitoring of pathogen circulation, seasonal trends, and emerging resistance mechanisms while supporting outbreak detection and public health responses.

Finally, economic benefits of syndromic testing include reductions in unnecessary antimicrobial therapy and avoidance of adverse drug events, fewer additional diagnostic procedures [110], shorter hospital length of stay and improved patient flow, earlier infection prevention interventions resulting in fewer unnecessary isolation days and reduced overall healthcare resource utilization [16,70,75]. Although multiplex molecular assays generally have higher per-test costs than conventional microbiology or single-target molecular assays, their economic value should be assessed within the broader context of overall healthcare utilization and potential downstream clinical and operational benefits rather than laboratory costs alone. Multiple studies have demonstrated that downstream savings may partially or completely offset the higher upfront costs of syndromic testing, particularly when these assays are implemented as part of comprehensive antimicrobial stewardship and infection prevention programs [70,75,99,111].

Cost-effectiveness may vary substantially across clinical syndromes and patient populations. Bloodstream infection and pneumonia panels have generally demonstrated the greatest economic benefit because of their ability to improve management of critically ill patients and reduce the costs associated with prolonged hospitalization. In contrast, the cost-effectiveness of respiratory and gastrointestinal panels depends more heavily on appropriate patient selection, institutional testing strategies, and diagnostic stewardship. Future reimbursement policies and value-based healthcare payment models will likely play important roles in determining the long-term adoption and sustainability of syndromic diagnostics.

## 4. Challenges in Clinical Utilization and Integration

The current high reagent costs, need for capital equipment investment, potential for over-detection (particularly of commensal organisms or pathogens of uncertain clinical significance), and the risk of missing off-panel organisms are among many factors that remain challenging in optimal utilization of syndromic testing panels in different clinical settings. Also, molecular detection of nucleic acids does not necessarily indicate active infection and may reflect colonization, asymptomatic carriage, prolonged shedding, or residual nucleic acids following successful treatment. These limitations underscore the necessity of appropriate test selection, specimen collection & handling, result interpretation, and integration of institutional diagnostic stewardship and antimicrobial stewardship [15,16,100].

The greatest clinical benefits of syndromic testing are consistently observed when rapid diagnostic information is integrated into antimicrobial stewardship programs. Among all syndromic applications, bloodstream infection panels have generated the strongest evidence supporting this principle. Rapid pathogen identification and resistance gene detection substantially shorten the time required to obtain actionable microbiological information. However, reductions in mortality, time to effective therapy, and healthcare utilization are most pronounced when stewardship teams actively review results and communicate therapeutic recommendations to clinicians [28,29]. Pneumonia and joint infections panels present particularly attractive ASP opportunities because they combine pathogen identification with resistance marker detection. Early recognition of AMR organisms may support timely escalation of therapy, while negative resistance marker results may facilitate earlier de-escalation. These applications exemplify the synergistic relationship between rapid diagnostics and antimicrobial stewardship.

Successful implementation and optimal utilization of syndromic testing require overcoming several technical, operational, and interpretive challenges associated with current testing platforms [15]. Realization of the full clinical value of syndromic testing requires sustained multidisciplinary collaboration to ensure appropriate test utilization, accurate result interpretation, timely clinical intervention, and effective integration of diagnostic information into patient management and public health practice.

The epidemiology of MDROs is continuously evolving, presenting a challenge for syndromic panel design and interpretation. Current BCID2, joint infection and pneumonia panels detect AMR genes representing the major resistance mechanisms at the time of panel development; however, emerging carbapenemase enzymes (e.g., *bla_GES_*, *bla_OXA-23_* and *bla_OXA-58_*), plasmid-mediated colistin resistance (*mcr-1* through *mcr-9*), extended-spectrum β-lactamases beyond *bla_CTX-M_*, and newer mechanisms such as fosfomycin resistance determinants are not incorporated in most current panels [12,112]. This creates a risk of false reassurance when an AMR gene is not detected but phenotypic resistance arises from an unlisted mechanism. Therefore, clinicians should be aware of potential genotype–phenotype discordance, organism- and mechanism-dependent interpretation, incomplete coverage of resistance mechanisms by current syndromic panels, the continuing need for conventional phenotypic AST when indicated, and the risk of inappropriate antimicrobial de-escalation based solely on the absence of a detected resistance determinant.

Also, the etiology of individual clinical syndromes is not static. For respiratory infections, the COVID-19 pandemic fundamentally shifted pathogen epidemiology, displacing seasonal influenza during 2020–2021, causing unprecedented RSV epidemics in 2022, and introducing SARS-CoV-2 as a persistent high-priority target, driving rapid panel updates across all manufacturers. For bloodstream infections, the global dissemination of *Candida auris*, a multidrug-resistant emerging pathogen absent from early BCID panel versions, highlighted the need for timely panel updates; *Candida auris* is now included in the GenMark ePlex BCID-FN Panel [113]. For meningitis/encephalitis, declining incidence of *H. influenzae* type b and *N. meningitidis* in vaccine-era populations has shifted the relative importance of other targets, while emerging viral encephalitis in tick-endemic regions (i.e., Powassan virus) remain outside current panel target sets.

Pathogen distribution and AMR gene prevalence vary substantially by region, influencing the clinical utility of panels developed primarily based on US and Western European epidemiology.

Syndromic panels are subject to both overutilization by ordering panels in low-probability clinical scenarios where positive results are more likely to represent incidental detection of colonizers or co-infections of uncertain significance, and underutilization in high-probability, time-sensitive scenarios where rapid results would most influence clinical management. The high sensitivity of multiplex PCR assays detects organisms at concentrations far below culture thresholds, including transient viremia, asymptomatic carriage, and post-treatment DNA remnants, creating a risk of overdiagnosis and unnecessary antibiotic treatment.

Off-label applications of syndromic panels have been investigated in selected clinical settings [114,115,116]. For example, the BIOFIRE Joint Infection Panel has been evaluated with non-synovial specimens, including whole blood, pleural fluid, abscess fluid, and tissue specimens [114]; however, evidence remains limited, and such applications require appropriate laboratory validation before clinical implementation.

Table 4 summarizes the clinical indications, scenarios, targeted patient populations and their expected clinical impact of currently available FDA-cleared syndromic testing panel.

Furthermore, the clinical value of syndromic testing depends not only on analytical performance but also on appropriate utilization and interpretation. The preponderance of evidence demonstrates that the clinical impact of syndromic panels is substantially amplified, and potential harm mitigated when integrated with robust institutional ASPs and diagnostic stewardship frameworks [119]. Diagnostic stewardship core interventions include: (1) defining clinical indications for each panel; (2) removing low-yield targets from reflex ordering (e.g., *C. difficile* from hospitalized patient GI panels); (3) restricting panel ordering to clinical scenarios where the result will change management; (4) providing real-time clinician education about result interpretation; and (5) monitoring utilization metrics and outcomes [100]. Without active ASP engagement, rapid diagnostic results may not alter clinical management: studies demonstrate that BCID panel results without concurrent ASP intervention have limited impact on time to antibiotic optimization [16,58].

Syndromic testing presents unique stewardship challenges because of its broad pathogen coverage and high analytical sensitivity. While comprehensive testing can increase diagnostic yield, indiscriminate utilization may lead to unnecessary healthcare costs, detection of clinically irrelevant organisms, and diagnostic confusion. The potential for overdiagnosis is particularly relevant for assays capable of detecting colonizing organisms, prolonged viral shedding, or low-level nucleic acid signals of uncertain significance. Therefore, institutions implementing syndromic testing must establish evidence-based utilization criteria that align testing strategies with specific clinical indications.

Appropriate patient selection and collection of high-quality clinical specimens remains one of the most important determinants of clinical utility. Respiratory pathogen panels, for example, may provide substantial benefit among hospitalized patients, immunocompromised hosts, and outbreak investigations, whereas routine testing of low-risk outpatients may offer limited incremental value compared with targeted testing strategies. Similar principles apply to gastrointestinal, meningitis/encephalitis, pneumonia, and bloodstream infection panels. Diagnostic stewardship programs should therefore incorporate clear ordering criteria, clinician education, and periodic utilization review to ensure that testing remains aligned with patient care objectives.

In addition, the successful implementation of syndromic testing increasingly depends on integration with electronic health records, laboratory information systems (LIS), and clinical decision support infrastructure. As the volume and complexity of diagnostic data continue to increase, automated interpretation and communication tools have become essential for ensuring that clinically relevant information reaches providers in a timely and actionable manner. Modern LIS platforms can support syndromic testing through syndrome-specific ordering pathways, duplicate testing prevention, automated result reporting, interpretive comments, reflex testing algorithms, and real-time notification systems. The transition from legacy laboratory information systems to integrated electronic health record platforms, such as Epic Beaker, over the past few years, has significantly advanced diagnostic stewardship in many U.S. clinical laboratories.

## 5. Conclusions and Future Developments

Syndromic testing panels represent a paradigm shift in clinical microbiology and infectious disease diagnosis, enabling simultaneous pathogen identification and AMR gene detection across a broad diagnostic spectrum within clinically actionable timeframes. Clinicians increasingly expect actionable microbiological results within hours rather than days, while healthcare systems seek diagnostic strategies capable of simultaneously improving patient outcomes, antimicrobial stewardship, infection prevention, and operational efficiency. Across syndromic testing applications, the strongest and most consistent evidence supports substantially reduced diagnostic turnaround time and earlier pathogen identification. Evidence also supports improvements in antimicrobial prescribing and infection prevention practices, particularly when rapid results are integrated with diagnostic and antimicrobial stewardship interventions. Effects on hospital length of stay and mortality are variable and appear to depend on the clinical syndrome, patient population, and implementation setting. Evidence for a mortality benefit, in particular, remains inconsistent, although the strongest evidence has been reported in patients with bloodstream infections when rapid molecular identification is combined with active antimicrobial stewardship [10,12,16].

Despite the fact that current syndromic panels remain constrained by predefined target menus, limited resistance characterization, and incomplete integration with broader clinical decision-making processes, utilization of syndromic panels will continue to expand as the commercial landscape broadens and as clinical guidelines increasingly incorporate rapid multiplex diagnostics into evidence-based management pathways.

Priority areas for future development of syndromic testing panels include: (1) expansion of existing syndromic testing panels with addition of common pathogens missing in current panels; (2) Development of a comprehensive tick-borne disease panel (i.e., covering *Borrelia burgdorferi*, *B. miyamotoi*, *Anaplasma*, *Ehrlichia*, *Rickettsia*, *Babesia*, and arboviral pathogens) and a reliable sexually transmitted infection (STI) panel suitable for both laboratory and point-of-care use to provide standardized, FDA-cleared alternatives to currently available laboratory-developed and commercial assays with variable analytical performance; (3) Direct-from-blood pathogen detection remains an important unmet need in bloodstream infection diagnostics. Emerging approaches seek to bypass blood culture enrichment through development of a new syndromic panel directly form blood specimens, detection of microbial cell-free DNA by metagenomic sequencing, highly sensitive digital PCR, and other culture-independent molecular strategies. These technologies may substantially shorten specimen-to-result time, although challenges related to analytical sensitivity, contamination, interpretation of low-level microbial signals, antimicrobial susceptibility characterization, cost, and clinical validation remain; (4) small-footprint, low-cost POCT panels with 2–5 targets for emergency department and ambulatory care deployment; (5) validated rapid tests differentiating viral from bacterial infection in acute care settings; and (6) accelerated regulatory pathways for incremental AMR gene target additions to existing cleared platforms.

The future of infectious disease diagnostics will likely extend far beyond current syndromic testing paradigms as emerging technologies are poised to further transform diagnostic and public health microbiology beyond conventional pathogen detection. Of these, metagenomic next-generation sequencing enables unbiased detection of bacteria, viruses, fungi, and parasites directly from sterile body fluids and deep tissues while simultaneously providing microbial identification and genotypic antimicrobial resistance prediction [120,121,122]. Real-time whole-genome sequencing of cultured multidrug-resistant organisms supports rapid genotypic susceptibility prediction, transmission analysis, and institutional and population-level surveillance of pathogens and antimicrobial resistance [6,123,124]. Rapid phenotypic antimicrobial susceptibility testing substantially shortens turnaround time from days to hours while preserving the comprehensive susceptibility information provided by conventional culture-based methods. Host-response diagnostics complement pathogen-based testing by assessing the patient’s immune response to infection and improving discrimination between bacterial and viral diseases [35,125]. Artificial intelligence and advanced clinical decision-support systems further enhance these technologies through automated result interpretation, antimicrobial resistance prediction, outbreak detection, and integration of laboratory results with electronic health records, imaging, pharmacy, and public health surveillance data to provide increasingly sophisticated, personalized diagnostic and therapeutic recommendations [7,126]. These approaches promise not only broader pathogen detection but also more comprehensive characterization of antimicrobial resistance, virulence factors, host–pathogen interactions, and disease severity. These innovations are likely to complement existing platforms and contribute to increasingly sophisticated diagnostic ecosystems. It is noteworthy that although NGS, metagenomic sequencing, digital PCR, and AI represent important emerging developments, their implementation in routine clinical microbiology laboratories of the US remains limited compared with established syndromic molecular platforms.

Importantly, faster microbiological results alone do not ensure improved patient outcomes. The clinical value of syndromic testing and emerging technologies depends on appropriate patient and test selection, accurate interpretation of results, and timely clinical actions triggered by those results. Integration with diagnostic and antimicrobial stewardship programs and clinical decision-support systems is therefore critical for translating rapid laboratory results into meaningful improvements in patient care. Future studies should evaluate not only analytical performance and turnaround time, but also clinician response to results, adherence to stewardship recommendations, changes in antimicrobial and infection prevention practices, and patient-centered and economic outcomes.

As molecular diagnostic capabilities continue to expand, clinical microbiology laboratories will remain central to this evolution, ensuring that technological innovation translates into meaningful improvements in patient care, public health, and global infectious disease prevention and control. Sustainable implementation of syndromic testing and new technologies also requires institutional and healthcare-system support, including appropriate reimbursement and resource allocation for clinical laboratories. Realization of the full clinical and public health benefits of current and future technologies will depend not only on continued technological innovation, but also on robust institutional infrastructure and sustained multidisciplinary collaboration among clinical microbiology laboratories, infectious disease specialists, antimicrobial and diagnostic stewardship programs, infection prevention and control teams, public health agencies, healthcare administrators, and industry partners. Together, these advances will shape the next generation of infectious disease diagnosis, treatment, surveillance, and prevention in the US and worldwide.

## Figures and Tables

**Figure 1:**
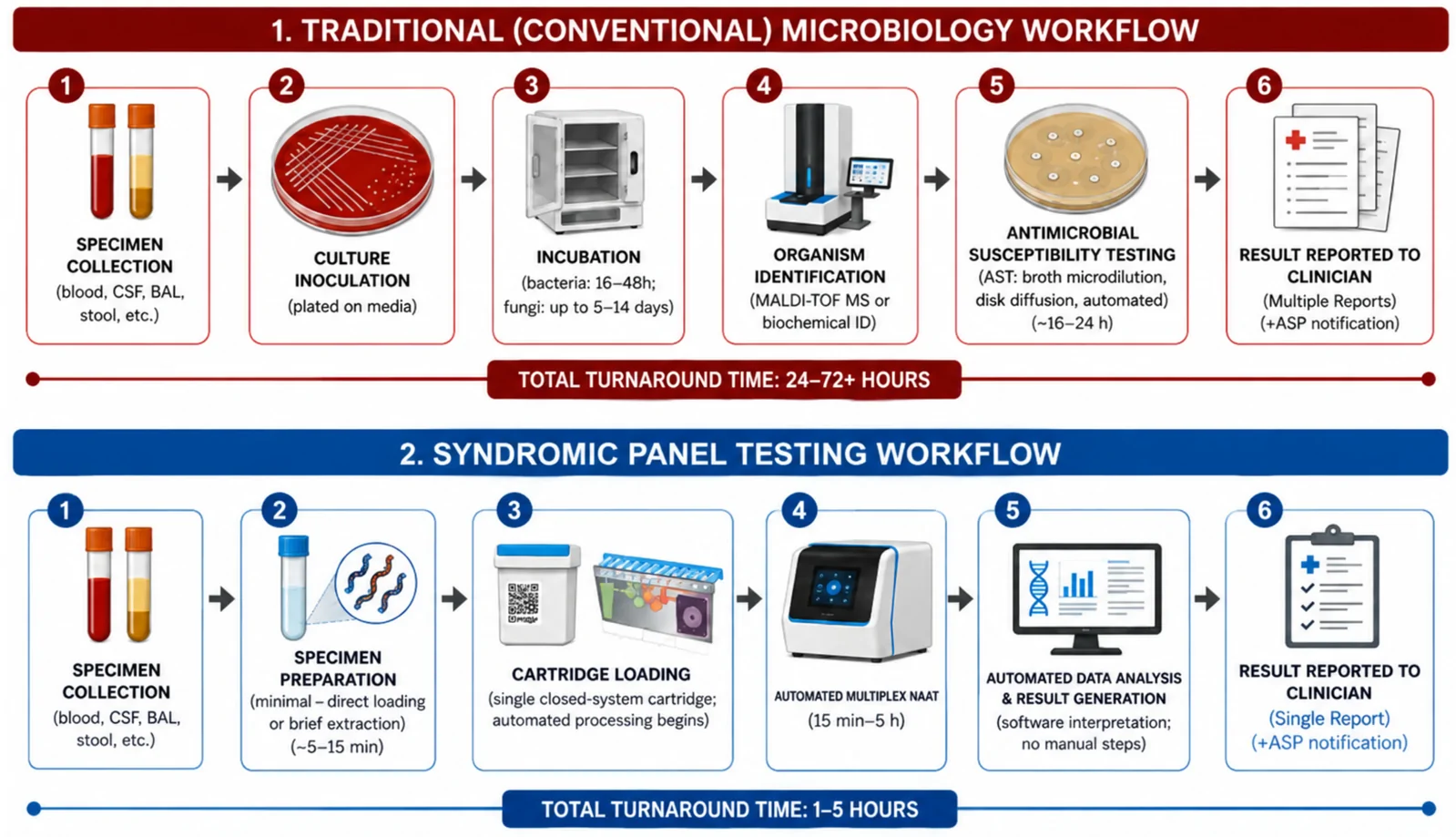
Comparison of conventional microbiology and syndromic panel testing workflows for infectious disease diagnosis. The figure is an original illustration prepared by the authors with AI assistance for graphical design. ASP, antimicrobial stewardship program; AST, antimicrobial susceptibility testing; BAL, bronchoalveolar lavage; CSF, cerebrospinal fluid. For bloodstream infection panels performed on positive blood cultures, the indicated TAT represents assay time after blood-culture positivity and does not include the preceding blood-culture incubation time.

**Table 2:** FDA-approved/cleared multiplex syndromic testing panels.

Syndrome	Manufacturer	Assay(s) *	No. of Targets	Target Breakdown	Specimen Type	Approx. TAT	FDA Status (Year) #
Respiratory	DiaSorin/Luminex	xTAG Respiratory Viral Panel (RVP)	12	12 viruses	NPS	8 h	K063765 & K081483 (2008)
		NxTAG-RPP, v2	21	19 viruses, 2 bacteria	NPS	4 h	K152386 (2015), K231758 (2024, v2)
	bioMérieux/BioFire	FilmArray Respiratory Panel (RP)	20	17 viruses, 3 bacteria	NPS	1 h	K103175 (2011), K123620 (2013)
		FilmArray Respiratory Panel 2.1	22	18 viruses, 4 bacteria	NPS	1 h	DEN200031 (2021)
		BioFire SPOTFIRE Respiratory/Sore Throat (R/ST) Panel	15	R: 11 viruses and 4 bacteria; ST: 10 viruses, 4 bacteria	NPS, throat	15 min	K232954 (2024)
	Roche/GenMark	ePlex Respiratory Pathogen Panel (RPP)	22	20 viruses, 2 bacteria	NPS	1.5 h	K163636 (2017) EUA202453 (2020)
	QIAGEN	QIAstat-Dx Respiratory SARS-CoV-2 Panel	21	18 viruses, 3 bacteria	NPS	70 min	K233100 (2024)
Pneumonia	bioMérieux/BioFire	FilmArray Pneumonia Panel	33	18 bacteria, 8 viruses, 7 AMR	BAL, sputum, TA	1 h	K180966 (2018)
	Curetis GmbH	Unyvero Lower Respiratory Tract (LRT)	30	19 bacteria, 1 yeast, 10 AMR	BAL, mini-BAL	5 h	K191967 (2019)
Bloodstream infection	bioMérieux/BioFire	FilmArray Blood Culture Identification 2 (BCID2) Panel	43	26 bacteria, 7 yeasts, 10 AMR	Positive blood culture	1 h	K130914 (2013)—BCID (27 targets) K193519 (2020)—BCID2 (43 targets)
	Roche/GenMark	ePlex BCID-GP/GN/FP Panels	Panel-specific	GP: 20 bacteria, 3 AMR; NP: 21 bacteria, 6 AMR; FP: 15 targets	Positive blood culture	1.5 h	GP: K181663 (2018) NP: K182619 (2019) & K213236 (2022) FP: K182690 (2018)
	DiaSorin/Luminex	Verigene Positive Blood Culture Test (BC-GP, BC-GN)	Panel- specific	GP: 12 Gram-positive, 4 AMR; NP: 8 Gram-negative, 6 AMR	Positive blood culture	2–3 h	GP: K113450 (2012) GN: K132843 (2014)
		LIAISON PLEX Yeast Blood Culture Assay	14	14 targets covering 15 yeasts	Positive blood culture	2 h	K240627 (2024)
	T2 BioSystems	T2Bacteria Panel	6	6 bacteria	Whole blood	3–5 h	K172708 (2018), K233184 (2024)
Gastrointestinal	bioMérieux/BioFire	FilmArray Gastrointestinal Panel	22	13 bacteria, 5 viruses, 4 protozoa	Stool	1 h	K140407 (2014)
		FilmArray GI Panel Mid	11	7 bacteria, 1 virus, 3 protozoa	Stool	1 h	K243885 (2025)
	DiaSorin/Luminex	xTAG Gastrointestinal Pathogen Panel	14	8 bacteria, 3 viruses, 3 protozoa	Stool	5 h	K140377 (2014), K191161 (2019)
		Liaison Plex Gastrointestinal Flex Assay	24	11 bacteria, 5 viruses, 8 parasites	Stool	2 h	K253722 (2026)
	Becton Dickinson and Company (BD)	BD MAX Enteric Panel (Viral, Bacterial, Extended Bacterial)	13	8 bacteria, 5 viruses	Stool	3.5 h	K170308 (2017), K181427 (2018)
	Serosep	EntericBio Dx Assay	7	5 bacteria, 2 protozoa	Stool	3 h	K182703 (2018)
	Applied BioCode	BioCode Gastrointestinal Pathogen Panel (GPP)	17	10 Bacteria 4 viruses, 3 protozoa	Stool	4 h	K190585 (2019)
	Qiagen	QIAstat-Dx Gastrointestinal Panel 2	16	8 Bacteria, 4 viruses, 4 protozoa	Stool, rectal Swab	70 min	K220062 (2024)
	Hologic	Panther Fusion GI (Bacterial, Viral, Parasite) Assays, 3 panels	16	4–8 bacteria, 4 viruses; 4 protozoa	Stool	2.5 h	K251868 (2025)
	Cepheid	Xpert GI Panel	11	8 bacteria, 1 virus, 2 protozoa	Stool	74 min	K251721 (2026)
CNS infection	bioMérieux/BioFire	FilmArray Meningitis/Encephalitis (ME) Panel	14	6 bacteria, 7 viruses, 1 yeast	CSF	1 h	K160462 (2016)
	Qiagen	QIAstat-Dx Meningitis/Encephalitis (ME) Panel	9	7 bacteria, 1 virus, 1 yeast	CSF	70 min	K242256 (2024)
Joint infection	bioMérieux/BioFire	FilmArray Joint Infection (JI Panel)	39	31 bacteria, 8 AMR	Synovial fluid	1 h	DEN200066 (2022)
Tropical/Global fever	BioFire Defense	BioFire Global Fever Special Pathogens Panel	16	4 bacteria, 8 viruses, 4 protozoa	Whole blood	1 h	K213362 (2023)
	BioFire Defense bioMérieux/BioFire	BioFire Global Fever Panel FilmArray Tropical Fever Panel	6	1 bacterium, 2 viruses, 3 protozoa	Whole blood	1 h	K220870 (2022) K243463 (2024)

* Only selected syndromic panels with 6 or more targets are listed, including the first FDA-cleared panel for each clinical syndrome and the most recent panels from each manufacturer. # For a complete and up-to-date list of FDA-cleared nucleic acid–based tests and 510(k) documents, refer to the FDA website: https://www.fda.gov/medical-devices/in-vitro-diagnostics/nucleic-acid-based-tests (accessed on 30 June 2026). FDA status: K for in vitro diagnostics (IVD or 510k), DEN for De Novo, EUA for emergency use authorization.

**Table 3:** Potential benefits associated with implementation of syndromic testing in clinical microbiology and infectious diseases.

Domain	Potential Benefits	Representative Outcomes
Diagnostic Stewardship	Rapid pathogen identification	Reduction in TAT from 24–72+ hours to 1–5 h
	Detection of multiple pathogens	Single test for bacterial, viral, fungal, and selected parasitic pathogens
	Detection of AMR markers	Early identification of key resistance determinants (e.g., *mecA*/*C*, *vanA*/*B*, CTX-M, KPC, NDM) to guide earlier targeted therapy
	Reduced dependence on culture	Improved detection of fastidious, nonviable, or difficult-to-culture organisms
	Workflow simplification, automation and standardization	Reduced hands-on time and manual processing steps, improved reproducibility and inter-lab consistency
	Improved laboratory efficiency	Consolidation of multiple tests into a single assay
	Earlier diagnosis	Rapid identification of etiologic agents and AMR markers leading to faster clinical decision-making
	Earlier initiation of targeted therapy	Reduced time to optimal antimicrobial treatment
	Reduction in unnecessary antimicrobial use	Improved antimicrobial selection and de-escalation
	Shorter hospital length of stay	Earlier diagnosis and treatment optimization facilitate discharge planning
	Improved patient outcomes and satisfaction	Reduced morbidity and mortality in selected patient populations
Antimicrobial Stewardship (ASP)	Optimized antimicrobial prescribing	More rapid initiation, escalation, de-escalation of targeted therapy; reduced broad-spectrum antimicrobial exposure
	Enhanced stewardship interventions	Timely ASP notification and targeted recommendations
	Reduced antimicrobial resistance pressure	Potential reduction in selective pressure associated with unnecessary antibiotic use
Infection Prevention and Control (IPC)	Rapid detection of transmissible pathogens and healthcare-associated clusters	Enhanced infection control practice by earlier implementation of isolation and measures to reduce nosocomial transmission and outbreak
	Enhanced surveillance capability	Real-time monitoring of circulating MDROs and emerging health threats to improve hospital emergency preparedness
Public Health	Earlier outbreak detection	Rapid identification of community and healthcare-associated outbreaks
	Improved disease and AMR surveillance	More complete and timely pathogen detection and reporting; enhanced monitoring of pathogen and AMR epidemiology; Improved tracking of seasonal and geographic trends
	Support for pandemic preparedness	Rapid deployment during outbreaks of novel pathogens
Financial and Operational	Potential overall cost savings	Economic benefits may offset higher assay acquisition costs by reducing sequential downstream testing, inappropriate antimicrobial use, shortening isolation and LOS
	Operational efficiency	More efficient utilization of laboratory, pharmacy, and clinical resources

**Table 4:** Clinical indications and expected clinical impact of FDA-cleared syndromic testing panels.

Syndromic Panel	Clinical Indications, Scenarios, and Targeted Patient Populations	Expected Clinical Impact	References
Respiratory Panel (RP)	Hospitalized patients with moderate-to-severe respiratory illness Immunocompromised, critically ill, elderly adults, and young children Respiratory outbreaks or seasonal epidemics Rapid diagnosis expected to guide antiviral/antibiotic use, isolation, admission, or cohorting	Earlier antiviral therapy Reduced use of antibiotics Improved isolation/cohorting Enhanced ASP	[2,10]
Pneumonia Panel (PN)	Suspected HAP/VAP Severe CAP requiring ICU admission Mechanically ventilated patients Immunocompromised or recent antibiotic exposure	Earlier targeted antibiotics Rapid AMR detection ASP intervention	[2,37,117]
Blood Culture ID (BCID)	Positive blood cultures Sepsis or septic shock ICU, transplant, neutropenic, or candidemia	Reduced time to optimal therapy Earlier de-escalation Potential LOS, mortality reduction	[2,58,59]
Gastrointestinal Panel (GI)	Severe/persistent diarrhea Bloody diarrhea, fever, dehydration or sepsis Immunocompromised or hospitalized patients Travel-associated diarrhea or outbreaks Not routinely recommended for uncomplicated diarrhea	Increased pathogen detection Earlier targeted therapy Outbreak recognition	[2,10,73]
Meningitis/ Encephalitis (ME)	Suspected meningitis/encephalitis undergoing lumbar puncture Neonates, children, immunocompromised, critically ill patients	Earlier pathogen identification Faster CNS-directed therapy	[2,10,85,118]
Joint Infection (JI)	Suspected septic arthritis Native joints and selected acute prosthetic joint infections Prior antibiotic exposure	Earlier surgical and antimicrobial management	[2,88,92]
Tropical/Global Fever (TF)	Acute undifferentiated fever after international travel Suspected imported/tropical infections Public health investigations	Earlier diagnosis Improved outbreak response	[2,96,97]

## Data Availability

No new data were created or analyzed in this study. Data sharing is not applicable to this article.
